# Haptic wearables as sensory replacement, sensory augmentation and trainer – a review

**DOI:** 10.1186/s12984-015-0055-z

**Published:** 2015-07-20

**Authors:** Peter B. Shull, Dana D. Damian

**Affiliations:** State Key Laboratory of Mechanical System and Vibration, School of Mechanical Engineering, Shanghai Jiao Tong University, Room 930, Mechanical Engineering Bld, 800 Dong Chuan Road, Shanghai, 200240 China; Boston Children’s Hospital, Harvard University, 330 Longwood Avenue, Boston, Massachusetts 02115 USA

**Keywords:** Rehabilitation, Impairment, Sensory feedback

## Abstract

Sensory impairments decrease quality of life and can slow or hinder rehabilitation. Small, computationally powerful electronics have enabled the recent development of wearable systems aimed to improve function for individuals with sensory impairments. The purpose of this review is to synthesize current haptic wearable research for clinical applications involving sensory impairments. We define haptic wearables as untethered, ungrounded body worn devices that interact with skin directly or through clothing and can be used in natural environments outside a laboratory. Results of this review are categorized by degree of sensory impairment. Total impairment, such as in an amputee, blind, or deaf individual, involves haptics acting as sensory replacement; partial impairment, as is common in rehabilitation, involves haptics as sensory augmentation; and no impairment involves haptics as trainer. This review found that wearable haptic devices improved function for a variety of clinical applications including: rehabilitation, prosthetics, vestibular loss, osteoarthritis, vision loss and hearing loss. Future haptic wearables development should focus on clinical needs, intuitive and multimodal haptic displays, low energy demands, and biomechanical compliance for long-term usage.

## Introduction

Sensory impairments, including somatosensory, vision, and audition loss can result from a spectrum of injuries and diseases such as limb loss, vision loss, and stroke and have long been known to reduce quality of life and prolong rehabilitation [1, 2]. As the world population ages, the magnitude of these problems will likely increase given the susceptibility to sensory impairments in older populations [3]. In the absence of treatments that completely restore natural sensory function, approaches focused on replacing or augmenting deficits may serve as effective alternatives.

Human skin has long been recognized as a receptor for communicating information [4]. Skin sensations such as pressure, vibration, and stretch can convey tactile messages that are carried to the brain via afferent nerves [5, 6]. For example, tactile feedback can be used to encode pressure and vibration measurements from a prosthesis to the skin of a user [7]. To train human movement, kinematics can be measured in real time and compared with predefined desired kinematics, and tactile feedback amplitude or frequency can then be modulated proportionally to error signals to alert users of desired changes [8–10]. Similarly, tactile feedback has been used to train repetitive movements such as swimming or gait [11–13] in which case feedback is initiated in periodic pulses instead of continuously. Another approach is the expert-trainee paradigm in which the expert performs movements, which are followed by the trainee via haptic feedback based on the kinematic errors between the expert and trainee [14].

Haptic wearables have the potential to address sensory impairments. We define haptics broadly as the sense of touch and includes vibration, texture, slip, temperature, pain, force and proprioception sensations. Smaller, lighter, and more powerful sensors, actuators, and processors have enabled a recent rise in wearable technology for clinical applications. Wearable systems have been used for performing home rehabilitation, assessing functional activity, detecting movement disorders, improving walking stability, and reducing joint loading [15–17]. These systems give users mobility and the freedom to perform normal tasks in natural environments.

**Fig. 1:**
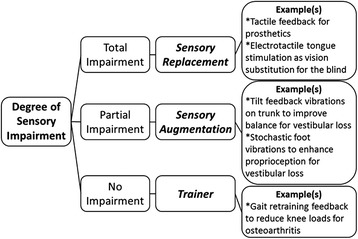
Haptic wearable applications classified by degree of sensory impairment

Clinical applications of haptic wearables may be classified by degree of sensory impairment (Fig. 1). Total impairment occurs when sensory function is completely lost, often resulting from damaged, dysfunctional, or missing sensory receptors or pathways such as for the blind and amputees. Total impairment requires sensory replacement either with the same sensing modality or as sensory substitution [18]. Incomplete sensory information may result from noisy, degraded sensory signals coincident with old age or the partial sensory loss from disease or injury. This leads to partial sensory impairment and can further affect function. For example, unilateral vestibular loss decreases postural control, which can lead to difficulties in standing or walking [19]. Haptic wearables may be useful for partial sensory impairment as a means of sensory augmentation facilitating motor control and rehabilitation [20]. In some clinical applications, sensory information remains intact but haptic wearables can be used to correct behavioral deficits such as retraining gait patterns to reduce knee loading for individuals with knee osteoarthritis. In this no impairment case, haptic feedback operates as a trainer, automatically guiding new movement patterns through cutaneous cuing information.

Due to recent rapidly increasing interest in wearables for clinical, research, and commercial purposes, there is a need to clearly present the state-of-the-art as it relates to impairments and rehabilitation. Thus, the purpose of this review is to examine haptic wearables for applications of varying degree of sensory impairment. While the focus was on portable devices, tethered devices demonstrating clinical benefits of wearable haptic feedback that could be made portable (e.g. battery-powered instead of outlet-powered) were also included. Wearable robotic rehabilitation or powered exoskeleton devices were not included as they have been the subject of previous review [21, 22]. The paper is organized by descending degree of sensory impairment beginning with sensory replacement, then sensory augmentation, and finally trainer.

## Sensory replacement

Haptic wearables can act as a sensory replacement for total impairments. This section covers haptic applications involving missing upper and lower limbs followed by vision and auditory loss.

### Upper-limb prosthetics

Prosthetic hands have achieved remarkable mechatronic capabilities (e.g. Revolutionizing Prosthetics and Otto Bock), however, up to 39 % of amputees wearing myoelectrically controlled prostheses do not use them regularly or at all due to a lack of tactile sensory feedback [23–26]. Current grasp information in prosthetic users occurs through visual observation (77 %), listening (67 %) and residual limb sensations (57 %) [27]. Haptics for total impairment aims to restore missing tactile or proprioceptive information vital to prosthetic grasp to prolong sustained prosthesis use [28–31]. A major challenge is orchestrating spatial and temporal stimulation patterns and energy demands such that they give rise to congruent neuronal representations of vibration, contact, force, pressure, slip or muscle impedance during long-term use.

**Fig. 2:**
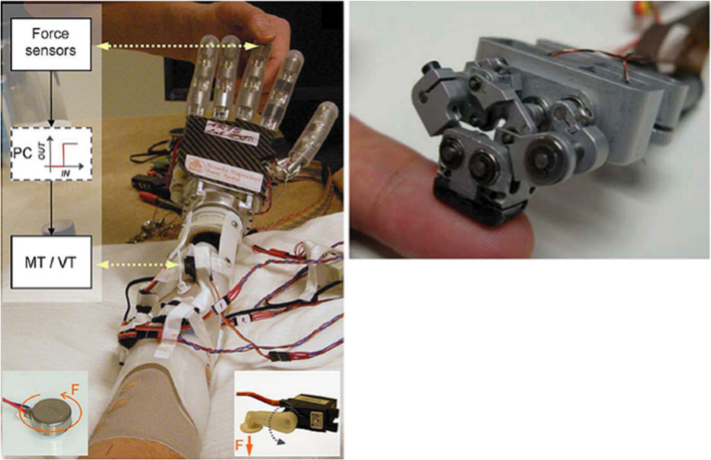
Haptic wearables for upper-limb prostheses. (left) Mechanical and vibroelectric haptic device for relaying pressure and vibration. Image from [35] used with permission from IEEE. (right) Compact wearable device for contact, pressure, vibration, shear, and temperature for amputees who underwent targeted nerve reinnervation surgery. Image from [47] used with permission from IEEE

Haptic feedback for upper limb prostheses restores the sense of touch by relaying force, pressure, and slip measurements to the user. Force and pressure feedback are commonly used in tactile devices to relay information about grip force. This information is typically transmitted mechanically, such as through skin tapping [32–35], or through electro- or vibro-stimulation [35–38] (Fig. 2 (left)). Patterson et al. [33] translated grip pressure from an object to hydraulic pressure in a cuff around the upper arm. By comparing combinations of pressure, vibration, and vision feedback, they found that pressure feedback resulted in the highest grasp performance. Rombokas et al. [39] found that vibrotactile feedback applied to the upper arm in force-motion tasks improved virtual manipulation performance for able bodied and prosthetic users.

Slip, or shear forces between prosthesis and object held, is pivotal for determining grasp stability and minimum grasp force [40–44]. Slip and force feedback in combination allow manipulation of a virtual object with lower forces than with force feedback alone [45]. Slip speed feedback, implemented as electrotactile stimulation on the skin, increases the success in stopping slip and regulates the user’s grip reaction time [46]. Kim et al. [47] built a tactile device for amputees after targeted nerve reinnervation surgery (Fig. 2 (right)). The device relays contact, pressure, vibration and shear through a mechanically-actuated tactor in contact with an 8 mm diameter patch of skin. Damian et al. [48] developed a wearable haptic device that relays slip speed, through a series of tactors that sweep across the skin and grip force through frequency-encoded tapping on the skin.

While many skin sites have been explored for tactile stimulation [49–52], fingertips are an attractive location due to the high density of the mechanoreceptors and the congruency of grasp sensation with the lost hand. Sites closest to the lost limb are preferred for the exploitation of redundant afferent terminals [35, 48, 53]. Other locations where skin sensation is used relatively less in normal life such as the arm or back have a lower density of mechanoreceptors but do not interfere with manipulative tasks [33, 52, 54]. However, it may be that the location of skin stimulation is less important than other factors such as learning rates [55].

Artificial motion proprioception allows prosthesis users to reach targets more accurately and reduces visual attention during manipulation [56, 57]. Witteveen et al. [58] used an array of eight vibrotactors on the arm to represent eight discrete positions in closing a prosthetic hand during grasping. Vibrotactile feedback was found superior to no feedback in grasp success and duration during virtual object grasping tasks. Bark et al. [6] introduce a wearable haptic device for rotational skin stretch to display proprioceptive limb motion. Users were able to discriminate rotational displacements of stretch within 6 degrees of the total range of motion. Artificial impedance feedback can support prostheses users to adapt the interaction of their prosthesis to a variety of environments. Blank et al. [59] showed that human users provided with position and force feedback are able to evaluate the effects of prosthesis impedance and its adjustability improves the users’ performance in minimizing contact forces with a moving object. In addition, vibrotactile [60] and skin stretch [61] have been used to provide users with the ability to regulate environment interaction forces.

These investigations show clear benefits of wearable haptic feedback for upper-limb prosthetics by restoring lost force, pressure, slip, and proprioception sensations. Current studies have primarily focused on restoring a single sensation, such as slip, while restoring multiple sensations simultaneously could endow users with more stable grasp and higher dexterity in real-life manipulation scenarios. A major challenge is miniaturizing bulky multi-function haptic wearables to a size where the benefits of the wearable device outweigh discomfort and inconveniences of complex devices which have thus far limited long-term user compliance.

### Lower-limb prosthetics

While a variety of lower limb prostheses exist, relatively few provide sensory feedback as compared to upper limb prosthetics [62]. However, the absence of feedback can lead to abnormalities in gait coordination, deficient balance, and prolonged rehabilitation [63–65]. To relay ground-to-prosthesis contact force information, Fan et al. [66] developed a tactile system consisting of a cuff of four silicone pneumatic balloons placed around the thigh that respond monotonically to pressure patterns recorded by force sensors in the insole of the user. Six healthy subjects were able to differentiate inflation patterns and direction of pressure stimuli, recognize three force levels and discriminate gait movements with 99.0 %, 94.8 %, 94.4 % and 95.8 % accuracy, respectively. Crea et al. [67] mapped the force recorded in the insole to vibrotactile feedback on the thigh skin, providing information about gate-phase transition. They demonstrated that the spatial and temporal relationships between vibrotactile time-discrete feedback and gait-phase transitions can be learned. In a study on twenty four transtibial prostheses users, Rusaw et al. [68] conveyed body motion through vibratory feedback proportional to signals from force sensors placed under the prosthetic foot. Vibratory feedback improved postural stability and reduced response time for avoiding falls. Proprioceptive feedback in lower-limb prostheses was investigated by Buma et al. [69] using a spatial electrotactile display of the prosthetic knee angle during gait. Subjects wore electrodes on the medial side of the thigh just above the knee, and the results showed that intermittent stimulation reduced habituation after 15 minutes. Finally, Sharma et al. [70] investigated the response in limb motion given vibration stimuli applied to the thigh, and showed that average response time was 0.8 sec, and response accuracy was greater than 90 %.

Most studies involving wearable haptics for lower-limb prosthetics have extracted various gait characteristics, such as foot pressure patterns or gait phase detection, from force-sensing insoles and then mapped these characteristics to prosthetic users via haptic feedback. While these initial studies are promising, future research should focus on restoring missing proprioceptive sensations at the ankle and knee joints in combination with foot pressure patterns.

### Vision aid for the blind

Engineers and scientists have long sought to enable visual substitution for the blind. In a seminal study, Bach-Y-Rita et al. [71] used a 20 x 20 array of tactors embedded in a dental chair to stimulate the skin of the back of blind subjects giving them a sense of “vision” through tactile substitution. Research built on these initial efforts has resulted in a host of haptic wearables as vision aids for the blind (see survey articles [72, 73]).

Although the waist has low tactile acuity, it is a natural location for haptic feedback as it moves relatively little during ambulation. McDaniel et al. [74] developed a tactile belt of 7 equidistantly spaced tactors around the waist to cue a blind user of another person’s presence. Results showed that the belt could convey another person’s direction via vibration location and another person’s distance via vibration duration. Karcher et al. [75] used a tactile belt consisting of 30 equidistantly spaced tactors in combination with a digital compass to display the direction of magnetic north by continually vibrating the closest tactor aligned with the magnetic north direction. Johnson and Higgins [76] used a tactile belt with two attached web cameras to convert visual information to a two-dimensional tactile depth map. Sensed objects triggered belt vibrations in the object’s direction, with closer objects causing higher vibration frequencies. Several studies have used tactile belts with GPS sensing for outdoor navigation by vibrating tactors in the direction of required movement to reach an intended waypoint or final destination [77–79].

**Fig. 3:**
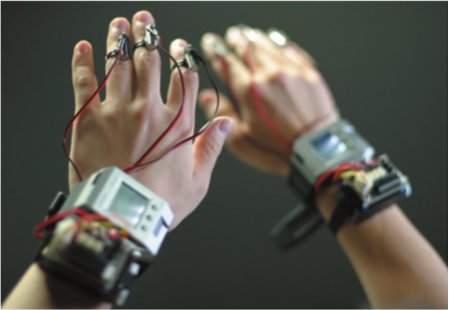
Wearable finger vibrotactors can be used to encode Braille characters and for guidance and navigation for the blind. Image from [80] used with permission from IEEE

The high density of mechanoreceptors in the hands and fingers make these good locations for haptic feedback. Amemiya et al. [80] attached vibrotactors to 3 fingers of each hand (Fig. 3) for guidance and navigation for the blind. Meers et al. [81] used electrostimulation gloves to relay tactile stimulation proportional to the distance to objects in the environment. Blindfolded subjects were able to report obstacle locations, avoid them, and walk to predefined destinations while navigating through outdoor locations including a car parking lot and college campus. Koo et al. [82] developed a soft, flexible fingertip tactile display with 20 electroactive polymer for Braille and displaying visual information through the skin. Shah et al. [83] created a cylindrical handheld tactile device with 4 ultrasonic sensors pointing front, left, right, and below the device held in front of the user. A 4 x 4 array of vibrotactors embedded in the handle aligned with the fingers grasping the device, with 4 tactors for each finger, excluding the thumb. Visual information from the ultrasonic sensors mapped to the tactors and enabled blindfolded subjects to navigate to a predefined location while avoiding obstacles. Ito et al. [84] created a handheld device tethered via a metal wire to the user’s belt. Users point the device in the direction of intended navigation, and when ultrasonic sensors detect objects, the wire tightens pulling the hand toward the belt. When objects are far away, the wire loosens allowing the hand to extend. Gallo et al. [85] equipped a white cane with tactile vibrators for distance feedback and a spinning inertia wheel to augment the contact sensation.

**Fig. 4:**
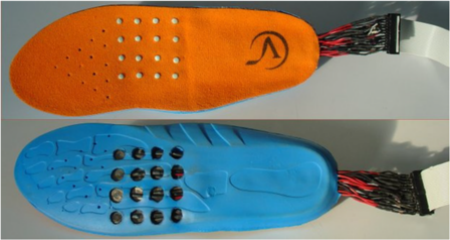
Vibration insoles can assist in navigation for the blind. Image from [89] used with permission from IEEE

Other locations targeted for haptic feedback as vision aids include the tongue, mouth, torso, head, and feet. Bach-Y-Rita et al. [49] developed a tongue stimulator composed of a 7 x 7 electrotactile elements. Users recognized tactile stimulation patterns including circles, squares, and triangles, which could potentially be used for blind navigation. Tang and Beebe [86] designed an oral tactile mouthpiece which stimulates the roof of the mouth via a 7 x 7 electrotactile display. The device delivers basic navigation direction cues including move left, right, forward, or backward. Jones et al. [87] used a 4 x 4 array of vibrotactors along the lower back to guide subjects through a grid of cones outside in a field. Mann et al. [88] retrofitted a helmet with a Kinect camera and a vibrotactile array around the forehead to display visual information haptically for applications of blind navigation. Finally, tactors have been embedded in insoles and used to give direction cues for navigation and to communicate an elevated risk of falling potential [89, 90] (Fig. 4).

There is a clear tradeoff between user comfort and density of feedback information when deciding on the location to apply haptic feedback as a vision aid. While applying tactile sensations to the waist or sole of the foot may be natural locations given that most people already wear belts and shoe insoles, stimulating high-density mechanoreceptor areas such as the mouth and fingertips enables higher resolution feedback that may more realistically convey visual information. A key emphasis moving forward should be identifying the most critical visual information for the blind and mapping this in an intuitive way to the users. Given that human response to visual information tends to be application specific, such as responding to non-verbal communication cues versus changing gait patterns to avoid an identified obstacle during navigation, haptic feedback strategies may also need to be application-specific instead of attempting to generalize all visual information.

### Auditory aid for the deaf

To hold conversations, the hearing impaired typically rely on visual or tactile cues, such as fingerspelling, lip reading, or Tadoma. Alternatively, tactile vocoders perform a frequency analysis of incoming auditory signals and display spectral information as stimulation on the skin of the hearing impaired [91, 92]. Saunders et al. [93] presented an abdomen belt of electrotactile stimulators encoding speech frequencies for speech recognition in profoundly deaf children (hearing loss of greater than 90 dB for 250 Hz sound frequencies). Improvement in speech production and intelligibility was observed after a 4-month exploratory study. Boothroyd et al. [94] showed that intonation can be more easily recognized using mechanical strokes on the skin implemented as an array of eight solenoids actuated depending on the pitch extracted from a microphone or accelerometer. A comparison between multichannel vibrotactile and electrical tactile stimulation for relaying sound frequency is presented in [95]. The two tactile display devices differed in stimulation modality (vibrotactile, electrotactile), location of stimulation (forearm, abdomen), and voice processing (with and without noise suppression). Results showed that both devices provide benefits beyond lipreading alone. Bernstein et al. [96] compared three vibrotactile vocoders on the forearm in normal and hearing-impaired subjects and found that greater resolution in the second formant region and linear output scaling led to significant improvements of sentence lipreading with vocoders.

Apart from speech recognition, it is also difficult for the hearing impaired to discriminate environmental sound. Reed et al. [97] demonstrated that normal hearing and profoundly deaf subjects equipped with a wearable spectral tactual aid are able to identify two bits of information in four 10-item sets of sounds. Furthermore, because it is difficult for the hearing-impaired to control voice pitch, it is challenging for them to maintain a stable tone while speaking or singing. Sakajiri et al. [98] developed a device of 64 piezoelectric vibrators arranged in rows of displacing pins that contact the user’s finger. The pins push onto the skin displaying the difference between user and target pitch. Two hearing-impaired subjects with knowledge and practice in music tested the device capability to aid their singing. The tactile display system reduces the average musical interval deviation to 117.5 cent (cent is a logarithmic unit of measure used for musical intervals), which is comparable to that of normal hearing children.

The inherent complexity of language and subject-to-subject differences raises serious challenges in developing highly effective haptic displays for auditory replacements. It may be more realistic for haptic feedback to supplement existing auditory activities such as supplementing lipreading to resolve ambiguous lip-read messages [96, 99]. Further research should integrate more sensed auditory modalities into wearable haptic technology, such as audio frequencies, voice aspiration, and temporal characteristics patterns. Further work to optimize voice signal filters to comply with subject-specific impairments could bring further benefits through haptic displays.

## Sensory augmentation

For partial sensory impairments, wearable haptics may provide complementary information to augment weak and noisy sensory signals. This section covers wearable haptics for improving standing balance, walking balance, and rehabilitation for varied conditions such as vestibular loss, Parkinson’s disease, and stroke.

### Standing balance

**Fig. 5:**
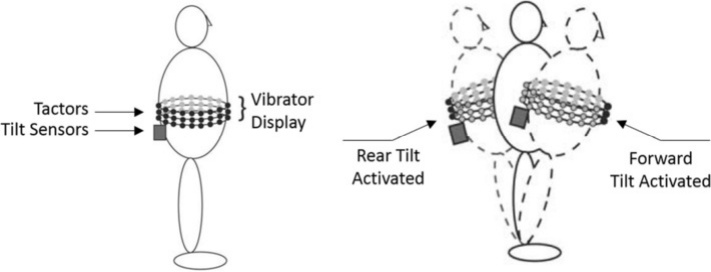
Tactor arrays can be used to improve standing posture through selective vibrations at the location needing correction. Image from [103] used with permission from IEEE

To improve balance for individuals with sensory impairments such as vestibular loss, researchers have focused on tactile feedback as sensory augmentation to reduce trunk sway [100, 101]. Wall et al. [102] showed that vibrotactile feedback applied to the sides of the trunk or shoulders could be used to reduce head-tilt angle and center of pressure displacements during standing posture with eyes closed. Subsequent testing showed that vibrotactor arrays placed around the waist could reduce anterior-posterior trunk tilt during quiet standing in individuals with vestibular deficits [101, 103]. Tactor vibrations cued subjects to move in the opposite direction of vibration (Fig. 5), and each tactor row indicated the severity of desired correction. Sienko et al. [104] found that 4 tactors spaced evenly around the waist were as effective at training trunk tilt as an array of 48 tactors (3 rows by 16 columns) placed around the waist. Jeka and Lackner [105] showed that touch and pressure stimulation at the fingertips can improve standing posture through the influence of apparent body orientation.

Vibrotactile sensations are typically used as a repulsive instructional cue (i.e. move away from the vibration) [103], though attractive instructional cues might be compatible with non-volitional responses to vibrotactile stimulation over certain anatomical regions [106, 107]. Haggerty et al. [108] tested the effect of the attentional load of vibration feedback by requiring subjects to perform a secondary task during standing posture vibration training. Ten healthy older adults performed standing balance training while simultaneously performing a secondary cognitive task (identifying a high or low pitched tone either verbally or by pressing one of two buttons). Subjects improved postural stability while performing a secondary task though their response times increased suggesting that vibrotactile feedback can be used to improve postural stability for older adults in cognitive loading situations. While tactile feedback is typically given based on trunk kinematic measurements, it has recently been suggested that incorporating muscle activation measurements in combination with kinematics may be more effective [109].

While haptic feedback for posture sway training is usually applied to the torso, the head and tongue are also suitable stimulation locations [110, 111]. Vuillerme et al. [112] used a 6 x 6 array (overall size of 1.5 cm × 1.5 cm) of electrotactile electrodes (1.4 mm diameter) to map foot center-of-pressure measurements to the tongue. The location of electrode stimulation corresponds to the location of the center of foot pressure thus augmenting each subject’s foot center-of-pressure perception. Tongue tactile feedback has been used for standing posture rehabilitation in individuals with unilateral and bilateral areflexia and unilateral and bilateral vestibular losses [113].

In contrast with previous studies utilizing haptic wearables as a cueing-based response for altering users of desired movement changes, stochastic resonance tactile vibrations have been suggested to amplify natural human afferent signals by adding white noise to a weak signal [114, 115]. Priplata et al. [116] used gel-based insoles with three embedded tactors to apply stochastic resonance white noise vibration to the sole of the foot. Twenty-seven elderly subjects stood quietly on insoles in conditions with and without input white noise. The amplitude of the noise was set to 90 % of the sensory perception threshold for each subject (and thus the noise signal was imperceptible during testing), and noise frequencies were 0–100 Hz. All standing balance metrics improved with stochastic noise. A similar study was performed showing stochastic resonance also improves standing balance for individuals with diabetic neuropathy and stroke [117].

Two primary strategies have emerged for applying wearable haptic feedback to augment standing balance: 1) apply periodic tactile cues, often to the torso, to instruct a desired corrective movement, and 2) apply continuous vibrations to the foot sole to amplify natural afferent signals. Combining these two methods could enable a superior system with greater potential to improve balance. Additionally, most studies assume wearable haptic devices need to be used indefinitely to continue providing balance aid benefits, while ignoring the effects of long-term learning and adaptation to such devices, which is a critical aspect deserving future consideration.

### Walking balance

Trunk movement in the medial-lateral plane is crucial for postural stability during gait [118]. Thus, research efforts have focused on providing tactile feedback to reduce excessive medial-lateral trunk movements. Dozza et al. [19] used a vibrotactile vest for gait training in nine subjects with unilateral vestibular loss. The vest contained two columns of three tactors on each side and pairs vibrated when medial-lateral trunk tilt exceeded 2 degrees (lower pair), 7 degrees (middle pair), and 12 degrees (higher pair). This training resulted in reduced trunk tilt, center of mass displacement, medial-lateral step width, and frequency of stepping error during gait. Horak et al. [119] performed two tactile feedback training sessions spaced two weeks apart in 10 individuals with unilateral vestibular loss. Feedback increased walking stability during tandem gait (heel-to-toe walking) evidenced by reductions in center-of-mass displacement, trunk tilt, and medial–lateral step width. Janssen et al. [120] tested 40 healthy subjects and showed that a vibrotactor visor utilizing tactile, visual, and auditory feedback reduced trunk tilt velocity and angles for a variety of gait tasks including walking: with eyes open or closed, while rotating or pitching the head, while carrying a glass of water, backwards, and up and down stairs.

Tactile feedback can increase attentional load during gait. Verhoeff et al. [121] observed 16 healthy young and 13 healthy old subjects as they performed gait training with a simultaneous secondary task, either walking while counting backwards in 7’s (cognitive task) or walking while carrying a tray with cups of water (motor task). Young subjects were able to perform both dual tasks, but elderly subjects could only perform the dual motor task and not the dual cognitive task. In gait retraining, continuous vibration feedback may be more appropriate than short periodic vibration pulses. Sienko et al. [122] tested seven subjects with vestibular loss who received either continuous vibration feedback of their trunk tilt angle or a periodic 200 ms vibration pulse immediately following heel strike on each step. While both methods enabled subjects to reduce medial-lateral trunk sway, continuous feedback was more effective.

Similar to applications in standing balance, stochastic resonance has been proposed as sensory augmentation to boost weak afferent signals for gait. Galica et al. [123] inserted three tactors into customized sandals to deliver 0–100 Hz white noise to 18 elderly recurrent fallers and 18 elderly non-fallers during 1 m/s walking gait. White noise foot vibrations reduced stride, stance, and swing time variability for elderly recurrent fallers and reduced stride and stance time variability for elderly non-fallers.

The benefits of wearable haptic feedback during gait must be weighed against the potential drawbacks. While tactile cues can help improve balance by reducing trunk sway, they also require additional cognitive attention that could result in negative secondary effects such as missing a curb while walking across a street. Future work should implement wearable haptic training systems that seek to minimize attentional load while maximizing gait improvements.

### Rehabilitation

For patients with neurological diseases, such as stroke, Parkinson’s disease, spinal cord injury, and peripheral neuropathy, haptic sensation is lost or distorted making everyday tasks difficult [124]. Artificial haptic feedback can play a role in regaining lost motor control [125]. Motor function improvement is achieved through task-oriented repetitive training during functionally related dynamic movements and the provision of artificial feedback [125, 126].

Upper extremity rehabilitation is often performed via vibrotactile feedback applied to the arm or hand to guide limb movements [8, 9, 36, 100, 127]. Jiang et al. [36] built a tactile wearable device to help multiple sclerosis patients improve grasp force during manipulation tasks by transmitting tactile information as a vibrotactile signal on the fingernail. Amplitude-based vibrotactile feedback was useful for patients with mild impairment in alerting them when grip force exceeded a predefined threshold. For those with severe impairment, better results were achieved by providing a feedback signal in which the frequency and duty cycle were proportional to the magnitudes of the contact forces. Lieberman et al. [8] developed a 5-DOF wearable robotic suit for improving human motion learning in rehabilitation. The suit was equipped with vibrotactile actuators placed near body joints which encoded arm postures. Tactile feedback provided by the suit yielded a 27 % improvement in accuracy while performing the target motion, and an accelerated learning rate of up to 23 %, compared to no feedback.

**Fig. 6:**
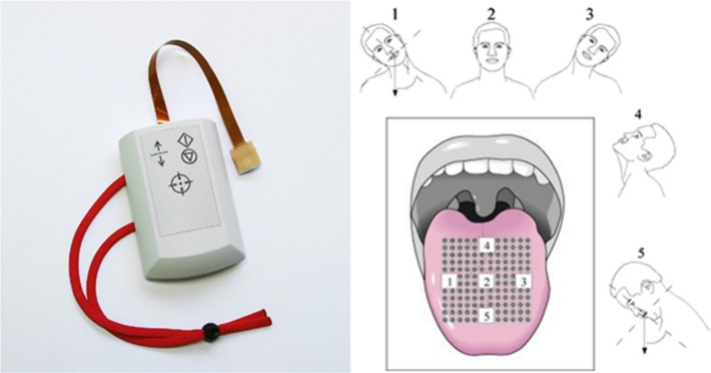
Sensory feedback applied to the tongue. (left) An electrotactile array for applying feedback to the tongue (Brainport balance device). (right) An example of tactile stimulation applied to the tongue to give feedback on head tilt for individuals with vestibular loss. Images from [132] used with permission from Elsevier

Haptic feedback for lower extremity rehabilitation is generally superior to standard therapy, placebo treatments, and verbal feedback for improving lower limb movements, and these benefits are generally maintained over time [128, 129]. Van Wegen et al. [130] presented a vibrotactile cueing device on the wrist to investigate whether Parkinson’s patients could adapt their stride frequency to rhythmic cues under conditions of changing walking speed and potentially distracting visual flow. Training resulted in lower stride frequency and was robust regardless of walking speed or visual distraction. Nanhoe-Mahabier et al. [111] demonstrated improved balance via a vibrotactile head-mounted display for twenty Parkinson’s disease patients. When trunk tilt exceeded a predefined threshold, vibration motors were activated in the direction of tilt to enable subjects to reduce trunk tilt. Peripheral neuropathy patients can improve postural instability and alter gait patterns via tactile feedback delivered as a two-segment ankle-foot orthoses in direct contact to the leg [128]. Gait rehabilitation was performed in peripheral neuropathy patients with sensory impairments on the bottom of the foot, with positive results increasing walking speed, step cadence or step length [131]. Insole pressure measurements were mapped to arrays of pneumatically-controlled silicone balloons on each ipsilateral thigh. In another study, twenty-nine patients with chronic balance impairments secondary to stroke were given tongue electrotactile feedback through a matrix of electrodes on the tongue (Fig. 6). The training was carried out 2 times per day 5 times per week for 1 week in the clinic, followed by 7 weeks as a home exercise program, which resulted in improvements in balance, balance confidence, gait function and quality of life [132].

While rehabilitation studies show increased performance with tactile feedback, a major disadvantage remains the variability between subjects, which impedes finding optimal feedback standards. Rehabilitation platforms capable of intelligent, adaptable tactile feedback configurations could provide subject-specific treatment more universally useful.

## Trainer

While most haptic trainer studies have not been clinically focused (e.g. drumming [133] or snowboarding [134] and jump landings [135]), increasing interest in haptic wearables makes this a likely area of growth. For example, haptic wearables can reduce knee loads by providing motion cues that alter risky walking patterns. One approach is to give subjects haptic feedback information directly related to knee loading and allow them to self-select a new gait pattern to reduce knee loads. Wheeler et al. [136] attached a single vibrotactor to the forearm which vibrated when knee loads exceeded a predefined threshold. No feedback was given when new gait patterns resulted in lower knee loads. Although effective in short-term, one drawback of this method is that subjects often self-selected awkward gait patterns that would likely not be maintained long-term.

Another approach is to explicitly train gait kinematics to reduce knee loading. Dowling et al. [137] embedded a pager motor inside a shoe to give vibration feedback to the foot based on lateral foot pressure. On each step subjects walked with lateral foot pressure above a predefined threshold, measured with a force-sensing resistor on the lateral underside of the shoe, the pager motor vibrated instructing a change in gait. Subjects quickly learned the medial foot pressure gait patterns, which resulted in significantly reduced knee loads. In other studies, vibration pulses on the lateral aspect of the shank just below the knee have been used to train individuals with knee osteoarthritis to internally rotate their toes by 5–7 degrees resulting in reduced knee loading and reduced knee pain over time [138, 139].

Training multiple kinematic parameters simultaneously [13] presents cognitive and motor challenges related to receiving and responding to multiple simultaneous channels of information. Lurie et al. [140] trained subjects to walk with new gait patterns involving kinematic changes to trunk sway, tibia angle, and foot progression angle by either giving error correction feedback cues on all parameters simultaneously or one parameter at a time. Perception accuracy was lower when all three vibrations were presented simultaneously on three consecutive steps as compared to one distinct vibration on each of the three steps. Subject performance was the same for all tactile feedback simultaneously and one feedback parameter at a time despite the fact that less feedback information was transmitted in the one feedback per step scenario. In another study, Jirattigalachote et al. [141] showed that when presenting multiple tactile feedback channels at separate skin locations simultaneously, subjects more accurately perceive different haptic stimuli (e.g. fast-adapting mechanoreceptor activation at one location and slow-adapting mechanoreceptor activation at the other location) compared to alike haptic stimuli during standing, walking, and jogging.

While haptic wearables have generally focused on treating existing problems, a shift in focus towards preventative medicine could enable a greater depth and impact in clinical applications. Knee osteoarthritis is one application in which tactile feedback has already been used to retrain gait movements to reduce knee loads that could potentially prevent the future development of osteoarthritis. Other future applications of wearable haptics as trainer could include correcting sitting posture to prevent back and neck injuries or correcting athletic movements to prevent ligament tears or bone fractures.

## Conclusions

For patients with total sensory impairment, haptic wearables can transmit missing information related to manipulation, walking, or speaking to complete the otherwise broken sensorimotor control loop. Motor disorders associated with partial sensory impairment have been addressed with haptic wearables that transmit behavioral cues, such as posture and gait guidance based on kinematic error signals in specific rehabilitation tasks. This same approach can be used for people with no sensory impairment to instruct movement changes to improve performance or prevent injury or disease. In addition to the specific suggestions for future work presented in each previous individual section in the body of this paper, we identified the following general design principles, based on the reviewed studies, important for developing future wearable haptic systems for sensory impairment:

*From need to practice.* A practical and efficient development of haptic wearables should follow a rigorous identification of the clinical requirements of the target condition. Haptic wearables must be collaboratively and comprehensively developed by involving clinicians, patients, scientists, and engineers, such that the devices are a product of clinical observations, direct end-user evaluation and feedback, up-to-date and integrative scientific knowledge and wearable technology [24, 25, 28].

*Bioelectrical/biomechanical compliance.* While various systems have been explored that demonstrate successful haptic mapping, further work is needed to develop mechanisms for long term efficacy and wearability, with special attention taken to comply with user kinematics, avoid user pain and fatigue, [142]. Reduced prosthesis weight has been found to be the highest priority design concern of prostheses users [25]. Miniature soft actuators [143–145] could ensure light haptic devices that do not impede the natural motions of the human body where they are mounted.

*Intuitive multimodal haptic representation.* The haptic representation of the transmitted information must be intuitive and easy to use [146]. Depending on the sensory impairment, haptic signals can display mechanics information (e.g. forces or angles) or instructional cues (e.g. desired movement change) encoded by signal magnitude, frequency or location on the skin. This pursuit becomes more challenging as multimodal feedback is integrated. Although most studies have only focused on a single modality, integrating multiple haptic modalities is necessary to comprehensively compensate for the missing sensation, e.g., force and slip feedback for upper extremity prosthetic manipulation, and limb position and planar pressure feedback for walking rehabilitation.

*Low energy demands.* Long term wearables rely on sustainable actuation and sensing. Novel energy sources and energy management should be considered in the design of the haptic device [147, 148]. For example, careful selection of power sources with high power-to-weight ratios and on-board computational algorithms to minimize power consumption could help meet these demands for tasks requiring extensive user training and long-term use.

*Long term usage.* Most haptic wearables are currently tested in short term tasks under laboratory conditions. Long-term testing is critical for developing and assessing sustainable haptic devices. This pursuit could significantly affect wearable device design and the implementation of feedback schemes and adaptive control algorithms to maintain the user performance over time.

One persistent question that repeatedly arose was, are haptic wearables best suited as temporary or permanent devices? Temporary devices can be used to train new movements which would eventually be internalized. Conversely, permanent feedback devices would be used indefinitely much like a prosthesis [109]. Horak et al. [119] showed that gait stability learning from biofeedback was not retained when the biofeedback was removed for a tandem gait task, and Dozza et al. [19] showed that a single session of practice with feedback did not result in lasting after-effects, which both indicate the need for either long-term training or permanent use. The duration of haptic wearables use may depend on the severity of the sensory impairment and the ability for long-term, sustainable motor learning in target populations. Ultimately, the fundamental goal of the haptic wearables is to assist sensory impairments in an unobtrusive manner, regardless of the severity of the user’s condition or length of treatment [149, 150].

**Fig. 7:**
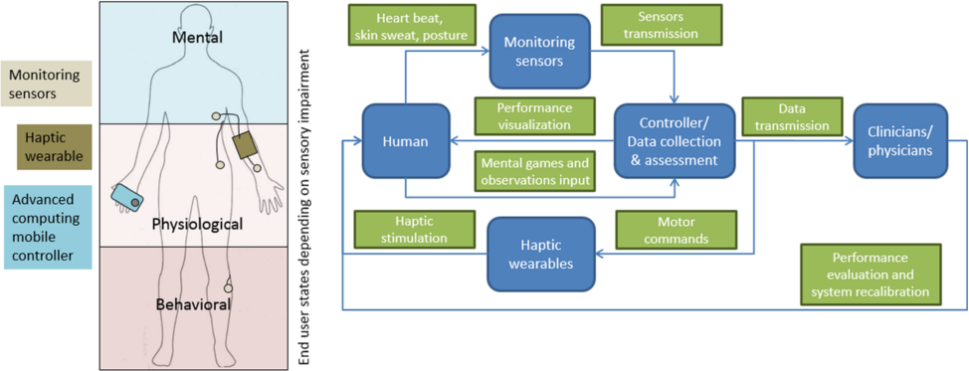
Future integrated haptic wearable systems. (left) Integrated haptic systems relay complete information about behavioral, physiological and mental state of users. (right) Advanced computing controllers regulate patient information processing and flow, transferring information to users and assistive staff

Future haptic wearables could incorporate mental, physiological, and behavioral measures (Fig. 7) to monitor health and appropriately adjust device functionality. Integrated haptic wearables could combine sensing of user's behavioral performance (e.g., manipulation tasks), physiological state (e.g. heart beat and electrodermal response sensing [151]), and cognitive state (e.g., questionnaire assessing cognitive ability) with a portable computing device, such as a smart phone.
